# Investigating the combined and unique contributions of positive psychological traits to sleep and exploring emotion regulation as a common mediator

**DOI:** 10.1007/s10865-023-00436-4

**Published:** 2023-09-12

**Authors:** Amber F. Tout, Donna C. Jessop, Eleanor Miles

**Affiliations:** School of Psychology, University of Sussex, BN1 9QH, Falmer, Brighton, UK

**Keywords:** Sleep, Mindfulness, Self-compassion, Gratitude, Optimism, Emotion regulation

## Abstract

The identification of variables which facilitate good quality and quantity sleep represents an important step in tackling the current global sleep loss epidemic. Previous research has established links between good sleep and the positive psychological traits of mindfulness, self-compassion, gratitude and optimism. However, studies have typically focused on single traits, limiting understanding of their collective and independent associations. The two studies reported here address this gap by exploring the combined and unique contributions of mindfulness, self-compassion, gratitude and optimism to sleep; Study 2 further investigated emotion regulation as a common underlying mechanism. Participants in both studies (Study 1 *N* = 268; Study 2 *N* = 333) completed online questionnaires assessing the four positive psychological traits and sleep quality and quantity; participants in Study 2 also completed measures of adaptive and maladaptive emotion regulation. Multiple regression analyses revealed that mindfulness, self-compassion, gratitude and optimism collectively accounted for 24.96% (Study 1) and 15.81% (Study 2) of the variance in overall sleep quality and quantity. Optimism and mindfulness emerged as significant linear predictors in their own right, with higher levels of optimism and mindfulness respectively being associated with better sleep. Study 2 further identified maladaptive emotion regulation as a common mediating mechanism. Findings highlight the importance of positive psychological traits in relation to sleep and indicate that optimism and mindfulness might make unique contributions to the prediction of sleep outcomes. Findings also flag emotion regulation as a potential common mediator of associations between positive psychological traits and sleep.

The importance of sleep for health is increasingly being recognised. Poor quality and quantity sleep has been identified as a risk factor for a wide range of adverse physical health outcomes, including coronary heart disease, stroke, diabetes, obesity, high blood pressure and – ultimately – all cause mortality (Cappuccio et al., 2010a, b, 2011; Ferrie et al., 2007; Guo et al., 2013; Miller et al., 2018). In addition, lack of sleep renders individuals more susceptible to mental health outcomes such as depression and anxiety (Cox & Olatunji, 2020; Zhai et al., 2015), exacerbates negative mood (Kahn et al., 2013; Konjarski et al., 2018) and increases the likelihood of developing neurodegenerative diseases including dementia (Shi et al., 2018; Xu et al., 2020).

Despite the overwhelming body of evidence documenting harmful consequences of inadequate sleep, many individuals obtain fewer than the recommended seven to nine hours a night (Chattu et al., 2019; Hafner et al., 2017; Sheehan et al., 2019). For example, a recent sleep survey in the UK indicated that 74% of individuals were getting less than seven hours sleep per night (The Sleep Council, 2017). Indeed, poor quality and quantity sleep is sufficiently prevalent throughout both developed and developing nations that researchers working in the field now consider it to be a global epidemic (e.g., Chattu et al., 2019; Stranges et al., 2012).

Identifying variables that facilitate good quality and quantity sleep has the potential to help address this sleep loss epidemic. One set of variables which shows promise in this regard relates to positive psychology, with existing research documenting links between higher levels of mindfulness, self-compassion, gratitude and optimism respectively and better sleep outcomes (e.g., Alkozei et al., 2019; Brown et al., 2021; Hernandez et al., 2020; Sala et al., 2020).

Mindfulness can be conceptualized as an increased level of acceptance and awareness of present moment thoughts, emotions and sensations, and a decreased tendency to engage in rumination and worry (Bishop et al., 2004; Ong et al., 2012). A recent meta-analysis supports the relationship between trait mindfulness and sleep, finding that higher levels of mindfulness were associated with better sleep outcomes (Sala et al., 2020). Moreover, further meta-analyses and reviews of experimental research indicate that mindfulness might play a causal role in impacting sleep, with results indicating that mindfulness interventions can lead to improvements in sleep (Ong & Moore, 2020; Rusch et al., 2019).

Self-compassion involves displaying kindness towards oneself in the face of suffering, accepting negative emotions as part of the common human experience, and being mindful towards painful feelings (Neff, 2003a). Recent meta-analyses have concluded that self-compassion is also associated with sleep. These analyses demonstrated both that higher levels of trait self-compassion were associated with better quality and quantity sleep (Brown et al., 2021; Butz & Stahlberg, 2020) and that experimentally manipulated self-compassion led to better sleep outcomes (Butz & Stahlberg, 2020), albeit only three experimental studies were included in this latter analysis.

While the research evidence linking dispositional gratitude and optimism respectively with sleep has yet to be subject to meta-analysis, there is also promising evidence that these traits might have important implications for sleep outcomes. Gratitude refers to the level of thankfulness that an individual experiences in relation to the positive outcomes they encounter in their day-to-day lives (Emmons et al., 2003). Several studies have reported links between trait gratitude and sleep, with individuals higher in gratitude experiencing better sleep (e.g., Alkozei et al., 2019; Wood et al., 2009). Furthermore, gratitude interventions have been shown to precipitate better quality and quantity sleep, strengthening the position that gratitude might play a causal role in influencing sleep outcomes (Boggiss et al., 2020; Emmons & McCullough, 2003, Study 3).

The literature is perhaps less clear-cut when considering evidence on the influence of optimism on sleep. Dispositional optimism is the general expectation that more good things will happen in the future than bad (Carver & Scheier, 2014). Multiple studies have identified links between higher levels of dispositional optimism and better sleep outcomes, both cross-sectionally and prospectively (e.g., Hernandez et al., 2020; Lau et al., 2017; Uchino et al., 2017). Moreover, there is some evidence that these relationships hold for objective measures of sleep (e.g., Lemola et al., 2011; but see also Hernandez et al., 2020), helping allay concerns that those higher in optimism might simply report or perceive better sleep irrespective of actual sleep quality and quantity. However, to the best of the authors’ knowledge, no published experimental studies to date have investigated the effects of optimism interventions on sleep outcomes, making it more difficult to ascertain whether optimism plays a causal role in this relationship. Nevertheless, there would seem to be sufficient evidence to consider trait optimism as a further plausible predictor of sleep.

In summary, a significant body of evidence associates each of mindfulness, self-compassion, gratitude and optimism with sleep. Critically, however, the research presented above has typically focused on links between a single positive psychology-related variable and sleep outcomes. Hence, there is little understanding of how the four positive psychological traits of mindfulness, self-compassion, gratitude and optimism collectively impact sleep and/or which trait(s) make significant independent contribution(s) to the prediction of sleep over and above their combined impact. Addressing these unresolved research questions has the potential to advance the positive psychology literature, by furthering our understanding of the combined and unique contributions of positive psychological traits to sleep outcomes. Moreover, ultimately, such knowledge could help guide the development of effective interventions, by identifying those positive psychology constructs most likely to make the largest unique contribution to sleep quality and quantity. Accordingly, the first aim of the studies reported in this paper was to investigate the combined and unique contributions of mindfulness, self-compassion, gratitude and optimism to overall sleep quality and quantity.

A further consequence of the tendency to focus on single positive psychological traits in sleep research is that little attention has been paid to investigating whether there might exist common underlying mechanisms linking these traits to sleep outcomes. Indeed, to date, research has primarily explored and established mediators specific to individual traits. For example, in the context of mindfulness, findings indicate that higher levels of mindfulness may benefit sleep because they are associated with lower levels of stress (Simione et al., 2020), less ruminative thinking (Liu et al., 2018), and a reduced likelihood of depressive symptoms and anxiety (Bogusch et al., 2016). In relation to self-compassion, it has been shown that this trait may facilitate good quality and quantity sleep via reductions in rumination (Butz & Stahlberg, 2018) and perceived stress (Hu et al., 2018). With regard to gratitude, findings suggest that higher levels of dispositional gratitude may exert positive effects on sleep by increasing positive (and decreasing negative) pre-sleep cognitions, which in turn reduces sleep-impairing thoughts and worries (Wood et al., 2009), and by lessening depressive mood state (Alkozei et al., 2019). Lastly, focusing on optimism, it has been shown that higher levels of this trait are associated with lower levels of anxiety and stress, which subsequently benefit sleep outcomes (Lau et al., 2017); furthermore, studies suggest that depressive symptoms and life satisfaction might also mediate the relationship between optimism and sleep (Lau et al., 2015; Uchino et al., 2017; but see also Lau et al., 2017).

We contend that a more thorough understanding of the associations between positive psychological traits and sleep may be gained by positioning them within a broader positive psychology – sleep framework, which unifies the literature on positive psychology and sleep by considering whether the same mediating mechanisms might explain links between the various traits and sleep outcomes. Specifically, we propose that emotion regulation represents a plausible common mechanism.

Emotion regulation refers to a level of “…awareness, understanding and acceptance of one’s emotions, as well as the ability to control and modulate behaviours in accordance with desired goals when experiencing negative emotions by using situationally appropriate strategies…” (Gratz & Roemer, 2004, p. 42). Emotion regulation strategies can be considered as adaptive (e.g., acceptance, positive reappraisal) or maladaptive (e.g., rumination, catastrophising) in nature due to their differential relationships with health and wellbeing outcomes (Garnefski et al., 2001). When it comes to sleep more specifically, adaptive strategies such as acceptance and positive reappraisal are generally linked to better sleep outcomes, whereas maladaptive strategies such as rumination and catastrophizing are related to higher levels of sleep-impairing arousal, insomnia and worse overall sleep quality and quantity (Cheng et al., 2020; Palmer et al., 2018).

Furthermore, previous research has demonstrated that constructs such as mindfulness, self-compassion and gratitude are linked to increased levels of engagement in adaptive emotion regulation and deceased levels of engagement in maladaptive emotion regulation (e.g., Boggio et al., 2020; Inwood and Ferrari, 2018; Roemer et al., 2015). In addition, many of the mediators previously implicated as underpinning relationships between the various positive psychological traits and sleep arguably relate to an individual’s (in)ability to regulate their emotions, for example: rumination (Butz & Stahlberg, 2018; Liu et al., 2018), stress (Hu et al., 2018; Lau et al., 2017; Simione et al., 2020), anxiety (Bogusch et al., 2016; Lau et al., 2017), depressive symptoms (Bogusch et al., 2016; Alkozei et al., 2019; Lau et al., 2015; Uchino et al., 2017) and pre-sleep thoughts and worries (Wood et al., 2009).

Indeed, researchers have recently started to investigate specific emotion regulation strategies as possible mediators of the associations between positive psychological traits and sleep. Thus, Semenchuk et al. (2022) demonstrated that self-blame (but no other emotion regulation strategy) mediated the association between self-compassion and sleep quality. However, to date, research has not explored whether adaptive and/or maladaptive emotion regulation might present a common mediator unifying associations between positive psychological traits and sleep.

In light of the above, we propose that emotion regulation represents a viable mediator which might underpin associations between these positive psychology-related constructs and sleep. Specifically, we contend that individuals with higher levels of trait mindfulness, self-compassion, gratitude and optimism will be more likely to engage in adaptive emotion regulation and less likely to engage in maladaptive emotion regulation which, in turn, will be associated with better quality and quantity sleep. Accordingly, as a second aim of the present research, in Study 2 we explore whether adaptive and /or maladaptive emotion regulation might mediate any associations between each of these positive psychological traits and sleep.

## Study 1

In line with the first aim of the present research, Study 1 investigated the combined and unique contributions of mindfulness, self-compassion, gratitude and optimism to sleep quality and quantity as assessed by the Pittsburgh Sleep Quality Index’s Global Sleep Score. It was hypothesised that, collectively, these positive psychological traits would account for a significant proportion of the variance in this sleep outcome. In addition, the opportunity was taken to investigate which (if any) of these positive psychological traits would make significant unique contributions to the prediction of sleep quality and quantity. No specific hypotheses were made regarding this more exploratory angle of the research, as – as described above - research to date has yet to consider the relative impact of these traits on sleep.

## Materials and methods

### Participants

Participants were recruited opportunistically, using social media and email, and invited to take part in a study about their thoughts, feelings and sleep. UK university departments were also contacted and asked to forward the information about the study to their students. Two hundred and sixty-eight participants completed the study and met the inclusion criteria that they did not work night shifts and did not have a diagnosed sleep disorder^1^. Ages ranged from 18 to 72 years (*M* = 32.63; *SD* = 14.45); 205 (76.49%) participants identified as female and 63 (23.51%) identified as male. The majority of the sample indicated that their nationality was British (82.46%), that their ethnicity could best be described as White (90.30%) and that their occupation could be categorised as either student (45.15%) or employed (41.42%).

### Design and Procedure

The study employed a cross-sectional, correlational design. The recruitment message circulated to prospective participants contained the web-link to the questionnaire, which was hosted by the online survey platform Qualtrics. In order to encourage participation, participants were given the opportunity to provide their contact details in a separate questionnaire if they wished to be entered into a prize draw with a chance to win one of two £25 vouchers. Participants provided informed consent before filling in the questionnaire and additionally gave permission for their data to be analysed upon its completion. The study was granted ethical approval by the appropriate body at the hosting university.

### Materials

The questionnaire included measures of the following constructs^2^:

**Demographic Information.** Participants were asked to indicate their age, gender, nationality, ethnicity and current occupation^3^.

**Mindfulness.** The 15 item Five-Facet Mindfulness Questionnaire (Baer et al., 2008), an established and validated measure (e.g., Gu et al., 2016), was used to assess mindfulness in the present study. Participants were asked to indicate how true each of the 15 statements was of them (e.g., “When I take a shower or bath, I stay alert to the sensations of water on my body”) on a 5-point Likert scale, ranging from *never or very rarely true* (1) to *very often or always true* (5). This measure had an acceptable level of internal consistency, α = 0.80, and a mean score was computed for each participant, with higher scores indicating higher levels of mindfulness.

**Self-Compassion**. Self-compassion was assessed with the Self-Compassion Scale (Neff, 2003a). This measure, which has been widely used and validated (e.g., Neff, 2016), comprises 26 items, e.g., “I try to be loving towards myself when I am feeling emotional pain” (*almost never* [1] to *almost always* [5]). This scale had an acceptable level of internal consistency, α = 0.94, and a mean score was calculated for each participant, with higher scores indicating higher levels of self-compassion.

**Gratitude**. The Gratitude Questionnaire Six-Item Form (GQ-6), which has been shown to have good psychometric properties (McCullough et al., 2002), was employed to assess participants’ tendency to experience gratitude in their daily lives. An example item is “I have so much in life to be thankful for” (*strongly disagree* [1] to *strongly agree* [7]). This measure was found to have acceptable internal consistency, α = 0.79, and a mean gratitude score was computed for each participant, with higher scores indicating higher levels of gratitude.

**Optimism.** Optimism was assessed using the Life Orientation Test – Revised (LOT-R), a validated measure (Scheier et al., 1994), which includes six items assessing overall levels of optimism; e.g., “In uncertain times I usually expect the best” (*strongly disagree* [1] to *strongly agree* [5]). This measure was found to have an acceptable level of internal consistency, α = 0.88, and a mean score was calculated for each participant, with higher scores indicating higher levels of optimism.

**Sleep Quality and Quantity.** Sleep quality and quantity were assessed via the Pittsburgh Sleep Quality Index (PSQI; Buysse et al., 1989). This scale includes 19-items assessing the following seven components of sleep quality and quantity over the previous month: subjective sleep quality, sleep latency, sleep duration, habitual sleep efficiency, sleep disturbances, use of sleep medication, and daytime dysfunction. Some of these items are open-ended questions (e.g. “During the past month, what time have you usually gone to bed at night?”), whereas others have fixed response scales (e.g. “During the past month how often have you had trouble sleeping because you cannot get to sleep within 30 minutes?”; *not during the past month* [0] to *three of more times a week* [3]). The resultant measure has been widely used and validated (e.g., Carpenter and Andrykowski, 1998). Responses to all items were scored in accordance with the PSQI manual to provide summary scores for each sleep component; possible component scores range from 0 to 3, with higher scores representing *worse* sleep in relation to each component. A global sleep score was subsequently calculated for each individual by summing their scores across the seven sleep components (internal consistency across the seven component scores was α = 0.77). Possible scores on the resultant global sleep score thus ranged from 0 to 21, with higher scores representing *worse* overall sleep quality and quantity.

## Results

### Preliminary analyses

Descriptive statistics for each of the positive psychological traits and sleep quality and quantity are summarised in Table 1, alongside bivariate correlations between these variables.

### Exploring associations between positive psychological traits and sleep quality and quantity

In order (a) to determine whether mindfulness, self-compassion, gratitude and optimism were collectively associated with sleep quality and quantity and (b) to explore which (if any) of these positive psychological traits made significant unique contributions to the prediction of this sleep outcome, a multiple regression analysis was conducted. Global sleep scores were regressed onto the four positive psychological traits. The resultant multiple regression analysis is summarised in Table 2.

Collectively, the positive psychological traits accounted for 24.96% of the variance in global sleep scores, *F*(4, 253) = 21.04, *p* < .001. Optimism and mindfulness emerged as significant linear predictors, with higher levels of optimism (β = − 0.32, *p* < .001) and mindfulness (β = − 0.19, *p* = .008) being associated with better sleep quality and quantity^4^.

**Table 1:** Descriptive Statistics and Bivariate Correlations for the Measures of Positive Psychological Traits and Sleep Quality and Quantity, Study 1

	2.	3.	4.	5.	Min.	Max.	*M*	*SD*	*n*
1. Mindfulness	0.65***	0.37***	0.53***	− 0.38***	1.00	4.73	3.10	0.56	268
2. Self-compassion		0.43***	0.68***	− 0.38***	1.31	4.65	2.91	0.73	268
3. Gratitude			0.60***	− 0.33***	2.00	7.00	5.73	0.89	268
4. Optimism				− 0.46***	1.00	5.00	3.22	0.82	268
5. Global sleep score					1.00	19.00	6.24	3.56	258

*** p < .001

*Note.* In accordance with the PSQI, lower scores are indicative of better sleep, hence negative correlations with positive psychological traits

**Table 2:** Summary of Multiple Regression Analysis Predicting Overall Sleep Quality and Quantity from the Positive Psychological Traits, Studies 1 and 2

	Study 1 Global Sleep Scores	Study 2 Global Sleep Scores
Mindfulness (β)	− 0.19**	− 0.18**
Self-compassion (β)	− 0.01	− 0.08
Gratitude (β)	− 0.07	− 0.10
Optimism (β)	− 0.32***	− 0.16*
Model *F*	21.04***	15.40***
Model *R*^2^	0.25***	0.16***

*** p < .001; ** p < .01; * p < .05

*Note.* Total *df* Study 1 = 257, Study 2 = 332

## Discussion

Results support the hypothesis that collectively the four positive psychological traits of mindfulness, self-compassion, gratitude and optimism would account for a significant proportion of the variance in sleep quality and quantity. These variables together accounted for almost 25% of the variance in this sleep outcome. In addition, the findings of Study 1 identified optimism and mindfulness as making significant unique contributions to the prediction of sleep quality and quantity, with higher levels of dispositional optimism and mindfulness being associated with better sleep.

## Study 2

In light of the replicability crisis in psychology and in the interests of open science, it is important both to demonstrate that findings are replicable and to pre-register confirmatory studies and corresponding hypotheses / planned analyses (Crüwell et al., 2019). Accordingly, the first objective of Study 2 was to explore whether the findings of Study 1 could be reproduced in a pre-registered study. In addition, the opportunity was taken to build on the findings of Study 1 by exploring emotion regulation as a possible mediator of the relationships between each of the positive psychological traits and sleep.

Study 2 thus represents a pre-registered replication and extension of Study 1. As with Study 1, the first aim of this study was to test the pre-registered hypothesis that, collectively, mindfulness, self-compassion, gratitude and optimism would account for a significant amount of the variance in sleep quality and quantity. To supplement this first aim, we again explored which (if any) of these positive psychological traits would make a significant unique contribution to this sleep outcome.

The second aim of Study 2 was to test the pre-registered hypothesis that emotion regulation would significantly mediate the relationships between each of mindfulness, self-compassion, gratitude and optimism and sleep quality and quantity. In light of the theory and research suggesting that adaptive and maladaptive emotion regulation strategies are likely to be orthogonal in nature (e.g., Garnefski et al., 2001), and thus could potentially have different implications for sleep, we elected to explore adaptive and maladaptive emotion regulation as independent constructs. Accordingly, the corresponding hypothesis was refined to specify that each of the positive psychological traits would be associated with greater engagement in adaptive emotion regulation strategies and less engagement in maladaptive emotion regulation strategies which, in turn, would be associated with better sleep.

## Materials and methods

The current study was pre-registered with the Open Science Framework: 10.17605/OSF.IO/43STP.

### Participants

A total of 333 psychology undergraduate students recruited from a university in the South of England took part in the present study and met the inclusion criteria that they did not work night shifts or have a diagnosed sleep disorder. Ages ranged from 18 to 49 years (*M* = 19.92; *SD* = 2.71); 282 (84.68%) participants identified as female, 49 (14.71%) identified as male, one (0.30%) identified as another gender and one (0.30%) elected not to report their gender. The majority of participants indicated that their nationality was British (78.98%) and identified their ethnicity as White (80.18%).

### Design and procedure

The current study employed a cross-sectional, correlational design. Participants were recruited via SONA – an online participant recruitment tool in which students sign up to studies in exchange for course credits – and invited to take part in a study about their thoughts, feelings and sleep. Participants who signed up to the study were given a link to the online questionnaire, which was hosted by the online survey platform Qualtrics. All participants provided informed consent electronically on the first page of the questionnaire and additionally gave permission for their data to be analysed upon completion; participants were compensated for their time with course credits. The study was granted ethical approval by the appropriate body at the hosting university.

### Materials

The questionnaire included measures of the following constructs^5^. Unless otherwise indicated, mean scores were computed for scales, with higher scores indicating higher levels of the construct in question.

**Demographic information.** Participants were asked to provide their age, gender, nationality and ethnicity.

**Mindfulness.** Mindfulness was again assessed with the FFMQ-15 (Baer et al., 2008), α = 0.73.

**Self-Compassion.** Self-compassion was assessed using the Self-Compassion Scale Short-Form (SCS-SF), a validated measure (Raes et al., 2011) comprising 12 items (e.g., “I try to be understanding and patient towards those aspects of my personality I don’t like”; *almost never* [1] to *almost always* [5]); α = 0.84.

**Gratitude.** Gratitude was again assessed with the GQ-6 (McCullough et al., 2002), α = 0.73.

**Optimism.** Optimism was again assessed using the LOT-R (Scheier et al., 1994), α = 0.80.

**Sleep Quality and Quantity.** Sleep quality and quantity was again assessed via the Pittsburgh Sleep Quality Index (PSQI; Buysse et al., 1989); internal consistency across the seven component scores in the present study was α = 0.67.

**Emotion Regulation.** Emotion regulation was assessed with the Cognitive Emotion Regulation Questionnaire (CERQ), which has been shown to have good validity and reliability (Garnefski & Kraaij, 2007). This scale includes a total of thirty-six items assessing nine emotion regulation strategies (five adaptive: acceptance, positive refocusing, refocus on planning, positive reappraisal, putting into perspective, and four maladaptive: self-blame, rumination, catastrophizing and other-blame). Participants were asked to indicate how they generally think in response to the negative or unpleasant events they experience (example positive refocusing item: “I think that I can become a stronger person as a result of the experience”; example catastrophizing item: “I keep thinking about how terrible my experience was”). Responses to all items were given on a 5-point Likert scale, ranging from *almost never* (1) to *almost always* (5). An overall adaptive emotion regulation score was computed for each participant by calculating the mean of the twenty adaptive emotion regulation items, α = 0.89. An overall maladaptive emotion regulation score was also computed for each participant by calculating the mean of the sixteen maladaptive emotion regulation items, α = 0.78.

## Results

### Preliminary analyses

Descriptive statistics and bivariate correlations between the measures of the four positive psychological traits, sleep quality and quantity and emotion regulation are given in Table 3. It can be seen that each of the positive psychological traits was significantly associated with adaptive emotion regulation, maladaptive emotion regulation and overall sleep quality and quantity. Furthermore, the measures of adaptive and maladaptive emotion regulation were significantly associated with overall sleep. It is also noteworthy that the correlation between adaptive emotion regulation and maladaptive emotion regulation was relatively low, thus justifying their treatment as orthogonal variables in our analyses.

**Table 3:** Descriptive Statistics and Bivariate Correlations Between the Measures of Positive Psychological Traits, Emotion Regulation and Sleep Quality and Quantity, Study 2 (N = 333)

	2.	3.	4.	5.	6.	7.	*Min.*	*Max.*	*M*	*SD*
1. Mindfulness	0.52***	0.30***	0.45***	0.42***	− 0.30***	− 0.32***	1.80	4.40	3.03	0.44
2. Self-compassion		0.33***	0.57***	0.61***	− 0.45***	− 0.29***	1.25	4.33	2.73	0.62
3. Gratitude			0.49***	0.34***	− 0.34***	− 0.26***	2.83	7.00	5.72	0.77
4. Optimism				0.39***	− 0.37***	− 0.33***	1.17	5.00	2.99	0.76
5. Adaptive emotion regulation					− 0.25***	− 0.17**	1.35	4.90	3.21	0.57
6. Maladaptive emotion regulation						0.31***	1.75	4.50	2.99	0.48
7. Global sleep score							0.00	18.00	7.15	3.03

*** p ≤ .001 ** p ≤ .01

*Note.* In accordance with the PSQI, lower scores are indicative of better sleep, hence negative correlations with positive psychological traits and adaptive emotion regulation

### Exploring associations between positive psychological traits and sleep quality and quantity

In order to address our first pre-registered hypothesis that, collectively, mindfulness, self-compassion, gratitude and optimism would account for a significant proportion of variance in sleep quality and quantity, global sleep scores were regressed onto the four positive psychological traits (please see Table 2). The resultant regression model indicated that mindfulness, self-compassion, gratitude and optimism together accounted for 15.81% of the variation in global sleep scores: *F*(4, 328) = 15.40, *p* < .001. Mindfulness (β = − 0.18, *p* = .004) and optimism (β = − 0.16, *p* = .022) again both emerged as significant linear predictors, with higher levels of mindfulness and optimism being related to better sleep quality and quantity.

### Exploring emotional regulation as a mediator of associations between positive psychological traits and sleep quality and quantity

**Fig. 1:**
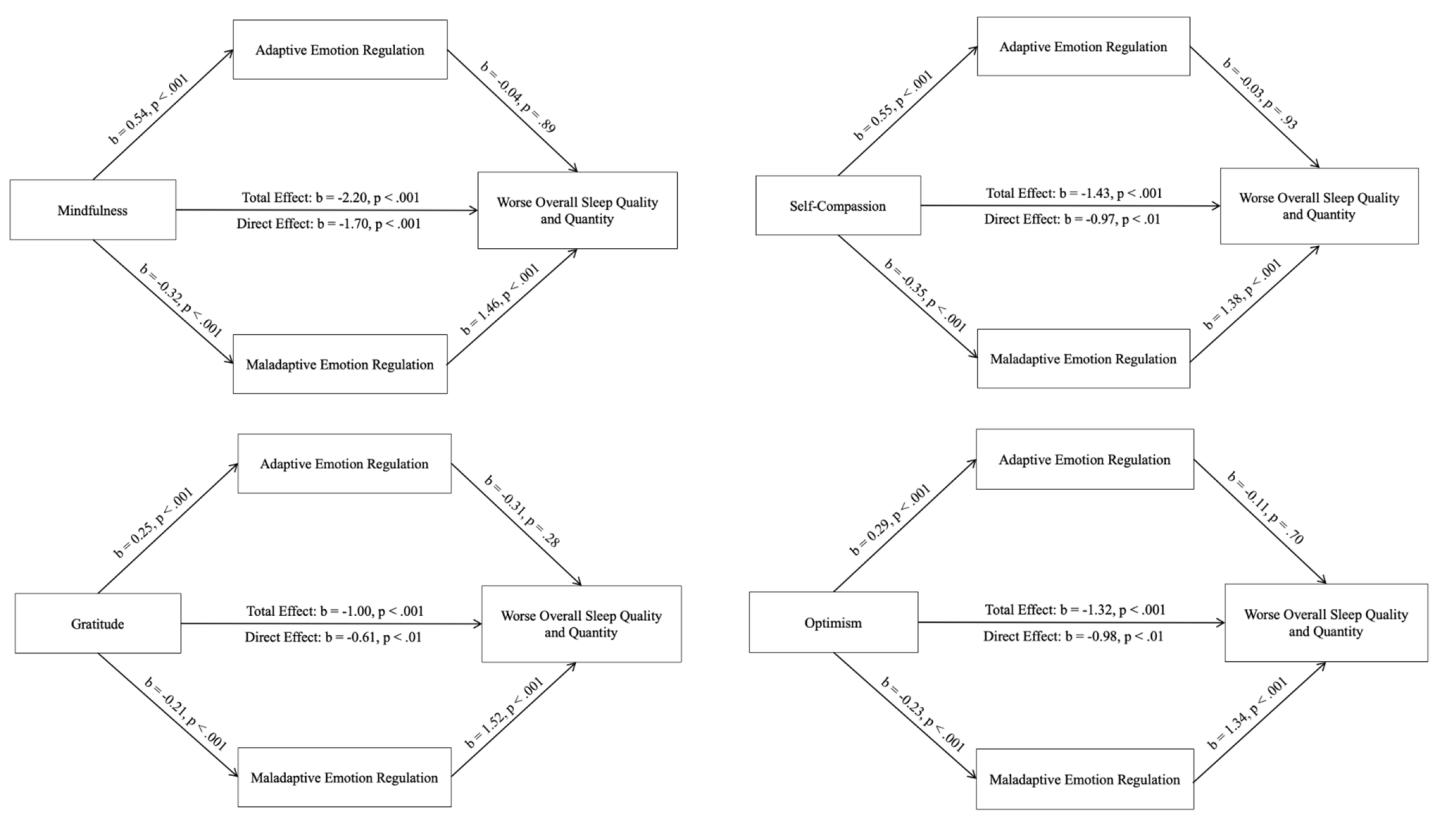
Mediation Analyses Displaying the Associations Between the Four Positive Psychological Traits and Sleep Quality and Quantity Through Adaptive and Maladaptive Emotion Regulation

To assess our second pre-registered hypothesis, that emotion regulation would significantly mediate the relationships between the four positive psychological traits (mindfulness, self-compassion, gratitude and optimism) and overall sleep quality and quantity, a series of mediation analyses were conducted using Hayes PROCESS for SPSS v3.5, taking 5,000 bootstrap samples to compute bias corrected confidence intervals, and adjusting for potential violations of heteroscedasticity (heteroscedasticity-consistent inference: HC2). For each of the four models, the positive psychological trait in question was entered as the predictor variable, global sleep scores were entered as the outcome variable, and adaptive emotion regulation and maladaptive emotion regulation scores were entered as the mediating variables. The resultant models are depicted in Fig. 1.

Analyses revealed that there was no significant^6^ indirect effect of mindfulness, self-compassion, gratitude or optimism on global sleep scores via adaptive emotion regulation (mindfulness: *b* = -0.02, BCaCI [-0.33, 0.26]; self-compassion: *b* = 0.17, BCaCI [-0.35, 0.39]; gratitude: *b* = -0.76, BCaCI [-0.23, 0.06]; optimism: *b* = -0.03, BCaCI [-0.20, 0.14]). By contrast, there were significant indirect effects of mindfulness, self-compassion, gratitude and optimism on global sleep scores via maladaptive emotion regulation (mindfulness (*b* = -0.47, 95% BCa CI [-0.75, − 0.24]), self-compassion (*b* = -0.48, 95% BCa CI [-0.74, − 0.24]), gratitude (*b* = -0.32, 95% BCaCI[-0.48, − 0.17]), and optimism (*b* = -0.31, 95% BCa CI [-0.49, − 0.15]). Thus, the impacts of mindfulness, self-compassion, gratitude and optimism on sleep quality and quantity were each partially mediated through maladaptive emotion regulation. In each case, higher levels of the positive psychological trait in question were associated with lower levels of maladaptive emotion regulation, which in turn was associated with better quality and quantity sleep.

## Discussion

The results of Study 2 provide further support for the hypothesis that the positive psychological traits would together account for a significant proportion of the variance in sleep quality and quantity, and again highlight optimism and mindfulness as making significant unique contributions to the prediction of this sleep outcome. Findings also identify maladaptive (but not adaptive) emotion regulation as a possible common mediator of the associations between these traits and sleep.

### General discussion

Results from both studies support the hypothesis that collectively the four positive psychological traits of mindfulness, self-compassion, gratitude and optimism would be significantly associated with sleep outcomes, accounting for a significant and sizeable proportion of the variance in sleep quality and quantity in both studies (approximately 25% and 16% respectively). These findings highlight the importance of positive psychological traits in relation to sleep and suggest that mindfulness, self-compassion, gratitude and optimism may collectively help facilitate good quality and quantity sleep. They also support, and start to integrate, the bodies of literature which have previously identified each of these traits as being independently associated with sleep outcomes (e.g., Alkozei et al., 2019; Brown et al., 2021; Hernandez et al., 2020; Sala et al., 2020).

It is interesting to note that the amount of variance in overall sleep quality and quantity accounted for by the positive psychological traits differed rather markedly between studies. We can only speculate as to why this might be the case; however, one possibility relates to the characteristics of the respective samples. For Study 2 the sample comprised solely of undergraduate students studying psychology (a degree programme with relatively few formal contact hours). Arguably, these students may have quite a lot of flexibility regarding their sleep patterns. For example, students who struggle to fall asleep or who wake during the night may simply be able to compensate by staying in bed longer the next day. As a result, it is plausible that their overall sleep quality and quantity might be less affected by variables that have the potential to influence sleep, such as positive psychological traits.

The results of our studies also provide some insight into which of the four positive psychological traits assessed in the present research might make the largest independent contribution to the prediction of sleep outcomes. Specifically, optimism and mindfulness emerged as significant linear predictors of overall sleep quality and quantity in their own right, such that greater levels of optimism and mindfulness were associated with better sleep. These findings thus suggest that optimism and mindfulness might make a unique contribution to sleep. It is noteworthy that the tables of correlations for both studies showed significant bi-variate correlations between each of the positive psychological traits and overall sleep quality and quantity. Given this, the findings of the regression analyses should not be interpreted to imply that optimism and mindfulness are the only two variables to be significantly associated with sleep outcomes or that they are the most important variables in this regard. Rather, these findings indicate that optimism and mindfulness make a significant unique contribution to the prediction of sleep over and above any shared variance between the various traits and sleep. It is perhaps unsurprising that some of the variability in sleep outcomes may be predicted by shared variance between the positive psychological traits, given that there are likely to be degrees of overlap and similarity between constructs such as - for example - mindfulness and self-compassion, where definitions of the latter include a mindfulness subcomponent (Neff, 2003a, b). Future research would benefit from trying to disentangle further the relative contributions of the different positive psychological traits (and perhaps their various subdimensions, where applicable) to sleep.

Ultimately, identifying the combined and unique contributions of positive psychological traits to sleep should allow these variables to be situated within a common theoretical framework, detailing their relative associations with sleep outcomes. Such a framework could then be used to inform the development of effective sleep interventions targeting such traits, in order to maximise their efficiency.

It is interesting that optimism emerged as significant linear predictor in its own right in the present studies, given the relative lack of research attention this individual difference variable has received as a potential target for intervention in the context of sleep. Indeed, this finding suggests that there may be some merit in experimentally manipulating individuals’ levels of optimism (e.g. Carrillo et al., 2019; Meevissen et al., 2011) and observing whether there are any attendant benefits for sleep. Such intervention studies would also help consolidate evidence suggesting that optimism may play a causal role in impacting sleep outcomes.

The present research also provides insight into *how* these positive psychological traits might impact sleep outcomes. Specifically, the findings of Study 2 indicate that maladaptive emotion regulation might represent a common mechanism underpinning the relationships between these traits and sleep. Mediation analyses thus indicated that there was a significant indirect association between each of mindfulness, self-compassion, gratitude and optimism and sleep via maladaptive emotion regulation. In each instance, higher levels of the positive psychological trait in question were associated with lower levels of maladaptive emotion regulation which in turn was associated with better quality and quantity sleep. Although the bivariate correlations indicated that the positive psychology traits were similarly associated with greater levels of adaptive emotion regulation and also that adaptive emotion regulation was associated with better sleep, the mediation analyses revealed no indirect impact of any of the four traits on sleep via this mechanism.

Although various mediators have previously been put forward and – on occasion – empirically tested for each of mindfulness, self-compassion, gratitude and optimism (e.g., Alkozei et al., 2019; Butz and Stahlberg, 2018; Simione et al., 2020; Uchino et al., 2017), studies have not previously attempted to establish whether there might be common mechanisms underpinning the impact of these four traits on sleep. The present research goes some way towards addressing this gap in the literature by identifying maladaptive emotion regulation as a potential underlying mediator between each of these traits and sleep. The identification of such common pathways between positive psychology-related constructs and sleep should ultimately allow for the development of common theories and frameworks which will help to unify the disparate strands of research on each of these traits and sleep outcomes.

It is noteworthy that, although there were significant indirect effects of mindfulness, self-compassion, gratitude, and optimism on sleep via maladaptive emotion regulation, the direct effects of each of these traits on sleep also remained significant. One explanation for this finding could be that there are additional underlying mechanisms linking these traits to sleep. Future research may thus wish to explore further common mediators, in order to better situate current findings within a broader framework.

The design of the present study is, of course, subject to limitations. The cross-sectional correlational design precludes us from drawing conclusions regarding causality, albeit previous experimental research in the domains of mindfulness, self-compassion and gratitude suggests probable causal paths from these traits to sleep (Boggiss et al., 2020; Butz & Stahlberg, 2018; Ong & Moore, 2020). Furthermore, the reliance on self-report measures of sleep is subject to inaccuracies introduced by, for example, the inability of individuals to accurately recall their sleep patterns over the previous month and/or social desirability biases. Although the PSQI is a reliable and validated measure (Buysse et al., 1989; Mollayeva et al., 2016), which has been widely used in studies exploring links between positive psychology constructs and sleep (e.g., Bogusch et al., 2016; Hu et al., 2018; Uchino et al., 2017; Wood et al., 2009), future research would nevertheless benefit from exploring whether the patterns of findings reported here hold for more objective measures of sleep outcomes (e.g., polysomnography or actigraphy; Marino et al., 2013). A further limitation to the present research relates to the samples used: the sample for Study 1 was recruited opportunistically, while Study 2’s sample was a student sample. The resultant samples are thus not representative of the general population; indeed, both samples were predominantly female, White, British, and relatively young. Such socio-demographic variables may well impact sleep patterns and hence limit the generalisability of our findings (e.g., Knutson, 2013). At a broader level, our studies are subject to the more general criticism that is levied at much research in in the behavioural sciences, in terms of its reliance on WEIRD (Western, educated, industrialised, rich, democratic) samples (e.g., Henrich et al., 2010). Future research should thus aim to establish whether the current findings hold across larger stratified samples drawn from a variety of populations.

In summary, the findings of both studies highlight the potential for the positive psychological traits of mindfulness, self-compassion, gratitude and optimism to collectively explain a significant amount of the variability in sleep quality and quantity. Future research should continue to explore their combined impact on sleep in order to more fully understand their shared and unique contributions to this fundamental health behaviour and to establish whether optimism and mindfulness consistently make independent contributions to the prediction of sleep outcomes. Such understanding could guide the development of more effective interventions to optimise sleep quality and quantity and – ultimately - help tackle the global sleep loss epidemic. The findings of Study 2 also identify maladaptive emotion regulation as a possible common mediator of the relationships between positive psychological traits and sleep. Establishing such shared pathways has the potential to help integrate research exploring associations between positive psychology-related constructs and sleep into one overarching theoretical framework, and future research would benefit from investigating further such common mechanisms.

## Data Availability

The authors confirm that the data supporting the findings of Study 1 are available within the supplemental online materials; the data that support the findings of Study 2 are available via the Open Science Framework.

## Footnotes

1 These criteria were implemented because individuals’ sleep patterns are likely to be substantially impacted by working night shifts and/or the presence of a sleep disorder. Accordingly, these factors could potentially confound any relationships between positive psychological traits and sleep.

2 The questionnaires included a number of additional measures. Only those measures relevant to the present research hypotheses are described here. Details of the full study materials are given at: 10.17605/OSF.IO/43STP. No other papers have been published from this data set and we have no current plans to publish any further papers from it.

3 Age, gender, nationality, ethnicity and current occupation were assessed to provide information about the characteristics of the sample and to establish how representative (or otherwise) the present sample was.

4 The scoring of the PSQI results in lower scores representing better sleep.

5 The questionnaire included a number of additional measures. Only those measures relevant to the present research hypotheses are described here. Details of additional measures are available at: 10.17605/OSF.IO/43STP. No other papers have been published from this data set and we have no current plans to publish any further papers from it.

6 The assessment of whether or not each indirect effect is statistically significant is based on inspection of the confidence intervals; where these do not cross zero the effect is considered to be significant. Please note, this approach (bootstrapping and inspection of confidence intervals) was used to test for mediation in preference to Sobel tests (which were specified in the pre-registration), as bootstrapping is considered to overcome various potential shortcomings associated with the Sobel test (please see Preacher and Hayes, 2008).
